# Unmet Support Service Needs and Health-Related Quality of Life among Adolescents and Young Adults with Cancer: The AYA HOPE Study

**DOI:** 10.3389/fonc.2013.00075

**Published:** 2013-04-08

**Authors:** Ashley Wilder Smith, Helen M. Parsons, Erin E. Kent, Keith Bellizzi, Brad J. Zebrack, Gretchen Keel, Charles F. Lynch, Mara B. Rubenstein, Theresa H. M. Keegan, AYA HOPE Study Collaborative Group

**Affiliations:** ^1^Outcomes Research Branch, Applied Research Program, Division of Cancer Control and Population Sciences, National Cancer InstituteBethesda, MD, USA; ^2^Department of Epidemiology and Biostatistics, School of Medicine, The University of Texas Health Science CenterSan Antonio, TX, USA; ^3^Human Development and Family Studies, University of ConnecticutStorrs, CT, USA; ^4^University of Michigan School of Social WorkAnn Arbor, MI, USA; ^5^Information Management ServicesRockville, MD, USA; ^6^Department of Epidemiology, The University of IowaIowa City, IA, USA; ^7^Children’s Hospital of MichiganDetroit, MI, USA; ^8^Cancer Prevention Institute of CaliforniaFremont, CA, USA

**Keywords:** support service needs, health-related quality of life, adolescent, young adult oncology, cancer

## Abstract

**Introduction:** Cancer for adolescents and young adults (AYA) differs from younger and older patients; AYA face medical challenges while navigating social and developmental transitions. Research suggests that these patients are under or inadequately served by current support services, which may affect health-related quality of life (HRQOL).

**Methods:** We examined unmet service needs and HRQOL in the National Cancer Institute’s Adolescent and Young Adult Health Outcomes and Patient Experience (AYA HOPE) study, a population-based cohort (*n* = 484), age 15–39, diagnosed with cancer 6–14 months prior, in 2007–2009. Unmet service needs were psychosocial, physical, spiritual, and financial services where respondents endorsed that they needed, but did not receive, a listed service. Linear regression models tested associations between any or specific unmet service needs and HRQOL, adjusting for demographic, medical, and health insurance variables.

**Results:** Over one-third of respondents reported at least one unmet service need. The most common were financial (16%), mental health (15%), and support group (14%) services. Adjusted models showed that having any unmet service need was associated with worse overall HRQOL, fatigue, physical, emotional, social, and school/work functioning, and mental health (*p*’s < 0.0001). Specific unmet services were related to particular outcomes [e.g., needing pain management was associated with worse overall HRQOL, physical and social functioning (*p*’s < 0.001)]. Needing mental health services had the strongest associations with worse HRQOL outcomes; needing physical/occupational therapy was most consistently associated with poorer functioning across domains.

**Discussion:** Unmet service needs in AYAs recently diagnosed with cancer are associated with worse HRQOL. Research should examine developmentally appropriate, relevant practices to improve access to services demonstrated to adversely impact HRQOL, particularly physical therapy and mental health services.

## Introduction

Advances in the diagnosis and treatment of cancer have increased the possibility of survival for many cancer patients. With nearly 69,200 adolescents and young adults (AYA) aged 15–39 diagnosed annually with cancer in the United States (National Cancer Institute, 2011), meeting survivorship care needs remains critical and understudied. Research on AYAs with cancer suggests that this population faces unique psychosocial and developmental needs when handling their healthcare. This is complicated by the fact that they encounter multiple social, emotional, and logistical challenges while simultaneously taking on new roles of responsibility and independence (Albritton and Bleyer, 2003; Fernandez and Barr, 2006; Zebrack, 2008). Unfortunately, there is also evidence that AYAs with cancer are under or inadequately served by existing support services (Zebrack et al., 2009, 2013; Dyson et al., 2012; Hall et al., 2012; Keegan et al., 2012).

Our study team previously found that 56 to 75% of AYA cancer survivors enrolled in the Adolescent and Young Adult Health Outcomes and Patient Experience (AYA HOPE) study who needed specific supportive care services including support group, pain management, physical or occupational therapy, mental health services, or financial advice on paying for health care did not receive these services (Keegan et al., 2012). Further, AYAs who were not currently in treatment (80%), reported that their physical health or emotional problems interfered with their social activities (a measure from the general health subdomain of the SF-12^®^) or had three or more physical symptoms were more likely to have an unmet service need (Keegan et al., 2012). In addition to a gap in support services, AYA cancer survivors have poorer cancer outcomes compared with pediatric and older adult patients (Stava et al., 2006), including poor health-related quality of life (HRQOL) (Zebrack et al., 2009; Clinton-McHarg et al., 2010; Smith et al., in press). Our study team additionally found that AYA cancer survivors exhibited significantly worse HRQOL across mental and physical health scales compared with the general population (Smith et al., in press). Survivors who were undergoing treatment, reporting current or recent symptoms, or lacking health insurance at any time since diagnosis were more likely to report worse HRQOL (Smith et al., in press). Though one Australian cohort study (Hall et al., 2012) has shown deficits in service and information needs as well as reduced HRQOL in AYAs with cancer, to date no studies have examined associations between service needs and HRQOL.

The current paper uses one of the largest population-based cohorts of AYAs diagnosed with cancer in the United States to examine relationships between unmet service needs across a variety of HRQOL domains including physical, emotional, spiritual, work/school, and financial outcomes. One of the main goals of the AYA HOPE study was to examine the impact of the cancer experience on health and psychosocial outcomes and to inform the development of future studies that focus on care and outcomes of AYAs. In the current study, we specifically examined relationships between any unmet service need and HRQOL as well as each individual unmet service need and HRQOL. This information will provide evidence to help understand the impact of unmet service needs on important health outcomes in the growing population of AYA cancer survivors.

## Materials and Methods

Details regarding AYA HOPE recruitment and study methods have been published previously (Harlan et al., 2011; Keegan et al., 2012). Briefly, respondents were diagnosed with a histologically confirmed non-Hodgkin lymphoma (NHL) (*n* = 121); Hodgkin lymphoma (*n* = 135); germ cell cancer (*n* = 193); acute lymphoblastic leukemia (ALL) (*n* = 16), or sarcoma (Ewing’s, osteosarcoma or rhabdomyosarcoma) (*n* = 19) between July 2007 and August 2009. Participants were age 15–39 years old at diagnosis, 6–14 months post-diagnosis at study entry, and able to read and write in English. Participants were recruited from one of seven National Cancer Institute (NCI) Surveillance, Epidemiology, and End Results (SEER) registries: Detroit, Seattle/Puget Sound, Los Angeles County, San Francisco/Oakland, Greater California, Iowa, and Louisiana. Study approval was obtained by each of the registries’ and NCI’s Institutional Review Boards. Of the 1,208 patients identified as eligible, 525 patients responded to the study (one respondent only consented to release of medical record data and one survey was lost, leaving 523 surveys), and medical records were obtained on 490 respondents. There was a 43% response rate for the overall study (Harlan et al., 2011).

In the AYA HOPE patient survey, respondents were asked questions about their demographic characteristics; barriers to and quality of healthcare; treatment and symptoms; insurance status, information and service needs, the impact of cancer, and HRQOL including psychosocial and physical functioning domains. Please see the following website for the study questions: http://outcomes.cancer.gov/surveys/aya/aya_hope_survey.pdf. The survey took approximately 15–20 min to complete.

### Measures

We describe the measures relevant to the current study aims below:

*Health-related Quality of Life*: Two instruments were used to assess physical and psychosocial functioning across the wide age/developmental range in the AYA HOPE study (Smith et al., in press). The PedsQL™ 4.0 was originally developed for use with children and adolescents, with a young adult version designed for individuals up to age 25 (Varni and Limbers, 2009) and validated in young adults with cancer (Ewing et al., 2009). It included the following domains: overall health, physical health summary, psychosocial health summary, emotional functioning, social functioning, and work/school functioning. We also included the PedsQL fatigue module (Varni and Limbers, 2008), given the importance of fatigue specifically for cancer patients. Further, as the PedsQL has only been validated up to age 25, we used the SF-12, version 2 (Ware et al., 2002), to best assess HRQOL outcomes in older young adults. The SF-12 has been validated for use in adults 18 and older and generates two global scores, using weighted subscale scores to compute the physical component summary (PCS) and mental component summary (MCS) scores (Ware et al., 2009).

*Service Needs*: Service needs included in this study were adapted from questions in a prior study of adult cancer survivors (Arora et al., 2007). Respondents were asked to indicate whether they had received (before, during, or after cancer treatment) the following supportive care services: participating in a support group; seeing a pain management expert; getting professional advice to help figure out payment for healthcare; seeing a physical or occupational therapist for rehabilitation; seeing a psychiatrist, psychologist, social worker, or mental health worker; talking with a spiritual or religious counselor about cancer; or having a nurse come to their home (Keegan et al., 2012). In addition, participants were asked whether they have needed (at the time of survey completion or now) need any of these services. A service need was considered unmet if the respondent did not receive the service, but reported needing the service (Keegan et al., 2012).

*Covariates*: Demographic data including age, sex, race/ethnicity, education level, and marital status, as well as health insurance (including whether respondents lacked health insurance at any time since diagnosis), and symptoms were collected from the self-report survey. Disease-related variables were obtained from SEER and medical records and included cancer type, AJCC stage, treatment type (surgery alone, radiation, chemotherapy or combined chemotherapy, and radiation), whether participants were receiving treatment at the time of the study, and comorbid conditions. Conditions in the comorbidity index had to be severe and/or chronic (i.e., serious and expected to affect treatment or outcomes or create significant health burden) and were categorized into condition groups and were summed to create final comorbidity scores as 0, 1, >2 based on a previously published comorbidity scale for AYA survivors (Parsons et al., 2012). Based on consultation with AYA oncology clinical experts, we included symptoms common to the cancer types being studied. Participants reported whether they experienced the following symptoms in the past four weeks: nausea/vomiting, frequent/severe stomach pain, diarrhea/constipation, pain in joints/bones, weight loss, weight gain, frequent/severe fevers, hot flashes, tingling/weakness/clumsiness of the hands/feet, frequent/severe headaches, frequent/severe mouth sores that impact eating/drinking, and problems with memory/attention/concentration. The number of symptoms was summed and to be consistent with our previous studies (Keegan et al., 2012; Kent et al., in press; Smith et al., in press). Symptom number was categorized as 0, 1–2, 3–4, or 5+.

### Statistical analysis

To examine associations between unmet service needs and HRQOL outcomes (PedsQL total, physical, emotional, social and work/school functioning scores, and fatigue scores and SF-12 physical and MCS scores), we used hierarchical multiple regression models controlling for factors found to be associated with HRQOL (Smith et al., in press), including age, sex, race/ethnicity, education, marital status, cancer type, stage at diagnosis, health insurance, current treatment status, treatment type, comorbidities, and symptoms. We examined models in which respondents indicated any, as opposed to no unmet service need, similar to other studies (Girgis et al., 2000; Sanson-Fisher et al., 2000; Keegan et al., 2012), as well as separate models with individual unmet service needs [service needs were uncorrelated with one another (all *r* < 0.50)]. Analyses were conducted using SAS version 9.2 software (SAS Institute Inc., Cary, NC, USA), and due to multiple comparisons, α was set at 0.01. Because 39 participants had missing responses, the analytic sample used in this analysis was *n* = 484.

## Results

Table 1 describes the demographic and clinical characteristics of the study participants. Approximately half of the respondents were 29 years of age or younger at diagnosis. The majority of participants were male, non-Hispanic white, working full time, and unmarried. Most participants had health insurance, but 14% reported lacking insurance at some point since diagnosis. Sixty-five percent of participants were diagnosed with early stage disease (stage I/II) and 82% were not in treatment at the time of study participation. Most participants (84%) had at least one symptom in the 4 weeks prior to completing the survey and 28% had a severe or chronic comorbidity.

**Table 1:** **Sample characteristics**.

	Unmet service needs			
	Total *n* (%)	None *n* (%)	Any *n* (%)	*p*-Value
Total	484	312	172	
**AGE AT SURVEY (YEARS)**				
15–17	21 (4.3)	18 (5.8)	3 (1.7)	*p* = 0.24
18–24	104 (21.5)	70 (22.4)	34 (19.8)	
25–29	114 (23.6)	71 (22.8)	43 (25.0)	
30–34	117 (24.2)	71 (22.8)	46 (26.7)	
35–41	128 (26.4)	82 (26.3)	46 (26.7)	
**SEX**				
Male	311 (64.3)	207 (66.3)	104 (60.5)	*p* = 0.20
Female	173 (35.7)	105 (33.7)	68 (39.5)	
**RACE/ETHNICITY**				
Hispanic	100 (20.7)	62 (19.9)	38 (22.1)	*p* = 0.24
White	285 (58.9)	193 (61.9)	92 (53.5)	
Black	43 (8.9)	23 (7.4)	20 (11.6)	
Other	56 (11.6)	34 (10.9)	22 (12.8)	
**EDUCATION (PRE-DIAGNOSIS)**				
High school or less	130 (26.9)	93 (29.8)	37 (21.5)	*p* = 0.05^∧^
Some college	127 (26.2)	72 (23.1)	55 (32.0)	
College graduate	226 (46.7)	146 (46.8)	80 (46.5)	
Missing	1 (0.2)	1 (0.3)	–	
**EMPLOYMENT STATUS (PRE-DIAGNOSIS)**				
Full time work	303 (62.6)	192 (61.5)	111 (64.5)	*p* = 0.74
Full time school	112 (23.1)	77 (24.7)	35 (20.3)	
Part time work	17 (3.5)	10 (3.2)	7 (4.1)	
Part time school	20 (4.1)	14 (4.5)	6 (3.5)	
Homemaker	15 (3.1)	9 (2.9)	6 (3.5)	
Unemployed/disabled	16 (3.3)	10 (3.2)	6 (3.5)	
Other/unknown	1 (0.2)	–	1 (0.6)	
**MARITAL STATUS**				
Married	205 (42.4)	132 (42.3)	73 (42.4)	*p* = 0.99^∧^
Not married	278 (57.4)	179 (57.4)	99 (57.6)	
Missing	1 (0.2)	1 (0.3)	–	
**LACKING HEALTH INSURANCE (ANY TIME SINCE DIAGNOSIS WITH NO COVERAGE)**				
No	407 (84.1)	274 (87.8)	133 (77.3)	*p* = 0.01^∧^
Yes	70 (14.5)	36 (11.5)	34 (19.8)	
Missing	7 (1.4)	2 (0.6)	5 (2.9)	
**CANCER TYPE**				
Acute lymphoblastic leukemia	16 (3.3)	11 (3.5)	5 (2.9)	*p* = 0.02
Germ cell cancer	193 (39.9)	137 (43.9)	56 (32.6)	
Hodgkin lymphoma	135 (27.9)	88 (28.2)	47 (27.3)	
Non-Hodgkin lymphoma	121 (25.0)	63 (20.2)	58 (33.7)	
Sarcoma	19 (3.9)	13 (4.2)	6 (3.5)	
**CANCER STAGE (AJCC)**				
Stage I	200 (41.3)	141 (45.2)	59 (34.3)	*p* = 0.03
Stage II	117 (24.2)	74 (23.7)	43 (25.0)	
Stage III	69 (14.3)	33 (10.6)	36 (20.9)	
Stage IV	59 (12.2)	36 (11.5)	23 (13.4)	
N/A	21 (4.3)	15 (4.8)	6 (3.5)	
Unknown	18 (3.7)	13 (4.2)	5 (2.9)	
**TREATMENT TYPE**				
Surgery only	59 (12.2)	43 (13.8)	16 (9.3)	*p* = 0.01
Radiation	50 (10.3)	42 (13.5)	8 (4.7)	
Chemotherapy	227 (46.9)	135 (43.3)	92 (53.5)	
Radiation and chemotherapy	108 (22.3)	66 (21.2)	42 (24.4)	
Missing/unknown/no treatment	40 (8.3)	26 (8.3)	14 (8.1)	
**IN CURRENT TREATMENT**				
No	396 (81.8)	258 (82.7)	138 (80.2)	*p* = 0.86^∧^
Yes	78 (16.1)	50 (16.0)	28 (16.3)	
Missing	10 (2.1)	4 (1.3)	6 (3.5)	
**COMORBIDITY**				
Missing	33 (6.8)	19 (6.1)	14 (8.1)	*p* = 0.03^∧^
0	315 (65.1)	212 (67.9)	103 (59.9)	
1	77 (15.9)	52 (16.7)	25 (14.5)	
2+	59 (12.2)	29 (9.3)	30 (17.4)	
**CURRENT/RECENT SYMPTOMS**				
0	78 (16.1)	64 (20.5)	14 (8.1)	*p* = < 0.0001
1 or 2	166 (34.3)	125 (40.1)	41 (23.8)	
3 or 4	108 (22.3)	59 (18.9)	49 (28.5)	
5+	132 (27.3)	64 (20.5)	68 (39.5)	
**MONTHS FROM DIAGNOSIS TO SURVEY**				
<10	131 (27.1)	77 (24.7)	54 (31.4)	*p* = 0.21^∧^
10–11	113 (23.3)	70 (22.4)	43 (25.0)	
12–13	122 (25.2)	81 (26.0)	41 (23.8)	
14+	117 (24.2)	83 (26.6)	34 (19.8)	
Missing	1 (0.2)	1 (0.3)	–	

*^∧^Missing responses not included in statistical analysis*.

More than one-third of AYAs reported at least one unmet service need (*n* = 172, Tables 2 and 3). The most commonly reported unmet service needs were financial (16%), mental health professional (15%), and support group (14%) services. Regression models showed that after adjusting for relevant demographic and medical covariates, having any (one or more) unmet service need was associated with lower HRQOL on all domains except the SF-12 PCS. Significant associations were found on the PedsQL total score, all PedsQL subscales: fatigue, physical functioning, emotional functioning, social functioning and school/work functioning, and the SF-12 MCS (all *p*’s < 0.0001; Tables 2 and 3).

**Table 2:** Multiple linear regression models* examining unmet service needs and PedsQL scales.

Unmet needs^#^	Total score							Fatigue				
	*N* = 484	%	*F*	*R*^2^	Beta	SE	*p*	*F*	*R*^2^	Beta	SE	*p*
*Any unmet service need*			16.10	0.59			<0.0001	13.48	0.51			<0.0001
No	312	0.64			–	–	–			–	–	–
Yes	172	0.36			**−9.21**	**1.42**	**<0.0001**			**−10.87**	**1.94**	**<0.0001**
*Individual unmet service needs*												
Nurse in home			15.29	0.54			<0.0001	11.75	0.47			**<**0.0001
No need	474	0.98			–	–	–			–	–	–
Unmet need	10	0.02			**−**0.51	4.67	0.91			**−**3.79	6.29	0.55
Support group			17.20	0.57			**<**0.0001	12.84	0.49			**<**0.0001
No need	417	0.86			–	–	–			–	–	–
Unmet need	67	0.14			**−10.67**	**1.94**	**<0.0001**			**−11.83**	**2.65**	**<0.0001**
Mental health professional			17.53	0.57			**<**0.0001	12.82	0.49			**<**0.0001
No need	410	0.85			–	–	–			–	–	–
Unmet need	74	0.15			**−10.69**	**1.80**	**<0.0001**			**−10.94**	**2.47**	**<0.0001**
Physical/occupational therapist			16.35	0.55			**<**0.0001	12.26	0.48			**<**0.0001
No need	440	0.91			–	–	–			–	–	–
Unmet need	44	0.09			**−9.74**	**2.39**	**<0.0001**			**−9.95**	**3.24**	**0.002**
Pain management expert			16.13	0.55			**<**0.0001	12.07	0.48			**<**0.0001
No need	442	0.91			–	–	–			–	–	–
Unmet need	42	0.09			**−8.78**	**2.41**	**<0.001**			**−**8.10	3.28	0.01
Spiritual/religious counselor			15.41	0.54			**<**0.0001	11.95	0.48			**<**0.0001
No need	453	0.94			–	–	–			–	–	–
Unmet need	31	0.06			**−**3.75	2.76	0.17			**−**7.33	3.71	0.05
Financial advice			15.36	0.54			**<**0.0001	12.01	0.48			**<**0.0001
No need	407	0.84			–	–	–			–	–	–
Unmet need	77	0.16			**−**1.93	1.88	0.31			**−**5.62	2.52	0.03

**Unmet needs^#^**	**Physical functioning**							**Emotional functioning**				

	***N* = 484**	**%**	***F***	***R*^2^**	**Beta**	**SE**	***p***	***F***	***R*^2^**	**Beta**	**SE**	***p***
*Any unmet service need*			13.68	0.51			**<**0.0001	7.65	0.37			**<**0.0001
No	312	0.64			–	–	–			–	–	–
Yes	172	0.36			**−6.28**	**1.87**	**<0.001**			**−12.01**	**2.01**	**<0.0001**
*Individual unmet service needs*												
Nurse in home			13.03	0.50			**<**0.0001	6.11	0.32			**<**0.0001
No need	474	0.98			–	–	–			–	–	–
Unmet need	10	0.02			**−**2.63	5.94	0.66			1.76	6.56	0.79
Support group			13.58	0.51			**<**0.0001	7.29	0.36			**<**0.0001
No need	417	0.86			**–**	**–**	**–**			**–**	**–**	**–**
Unmet need	67	0.14			**−7.82**	**2.53**	**0.002**			**−14.31**	**2.74**	**<0.0001**
Mental health professional			13.26	0.50			**<**0.0001	8.07	0.38			**<**0.0001
No need	410	0.85			**–**	**–**	**–**			**–**	**–**	**–**
Unmet need	74	0.15			**−**4.77	2.37	0.04			**−16.87**	**2.50**	**<0.0001**
Physical/occupational therapist			13.58	0.51			**<**0.0001	6.59	0.33			**<**0.0001
No need	440	0.91			**–**	**–**	**–**			**–**	**–**	**–**
Unmet need	44	0.09			**−9.48**	**3.06**	**0.002**			**−11.27**	**3.37**	**<0.001**
Pain management expert			13.63	0.51			**<**0.0001	6.35	0.32			**<**0.0001
No need	442	0.91			**–**	**–**	**–**			**–**	**–**	**–**
Unmet need	42	0.09			**−9.93**	**3.08**	**0.001**			**−**8.01	3.42	0.02
Spiritual/religious counselor			13.15	0.50			**<**0.0001	7.00	0.35			**<**0.0001
No need	453	0.94			–	–	–			**–**	**–**	**–**
Unmet need	31	0.06			5.34	3.50	0.13			**−17.27**	**3.79**	**<0.0001**
Financial advice			13.07	0.50			**<**0.0001	6.29	0.32			**<**0.0001
No need	407	0.84			–	–	–			**–**	**–**	**–**
Unmet need	77	0.16			2.31	2.39	0.33			**−**5.42	2.63	0.04

**Unmet needs^#^**	**Social functioning**							**Work/school functioning**				
	***N* = 484**	**%**	***F***	***R*^2^**	**Beta**	**SE**	***p***	***F***	***R*^2^**	**Beta**	**SE**	***p***

*Any unmet service need*			9.77	0.43			**<**0.0001	8.09	0.38			**<**0.0001
No	312	0.64			–	–	–			–	–	–
Yes	172	0.36			**−8.92**	**1.76**	**<0.0001**			**−9.91**	**1.99**	**<0.0001**
*Individual unmet service needs*												
Nurse in home			8.53	0.39			**<**0.0001	6.99	0.35			**<**0.0001
No need	474	0.98			–	–	–			–	–	–
Unmet need	10	0.02			**−**0.62	5.67	0.91			2.86	6.43	0.6567
Support group			9.24	0.41			**<**0.0001	7.80	0.37			**<**0.0001
No need	417	0.86			**–**	**–**	**–**			**–**	**–**	**–**
Unmet need	67	0.14			**−9.21**	**2.40**	**0.0001**			**−11.61**	**2.71**	**<0.0001**
Mental health professional			9.58	0.42			**<**0.0001	8.42	0.39			**<**0.0001
No need	410	0.85			**–**	**–**	**–**			**–**	**–**	**–**
Unmet need	74	0.15			**−10.32**	**2.22**	**<0.0001**			**−14.09**	**2.48**	**<0.0001**
Physical/occupational therapist			9.02	0.41			**<**0.0001	7.29	0.36			**<**0.0001
No need	440	0.91			**–**	**–**	**–**			**–**	**–**	**–**
Unmet need	44	0.09			**−9.31**	**2.92**	**0.002**			**−8.77**	**3.32**	**0.009**
Pain management expert			9.18	0.41			**<**0.0001	7.18	0.35			**<**0.0001
No need	442	0.91			**–**	**–**	**–**			**–**	**–**	**–**
Unmet need	42	0.09			**−10.75**	**2.93**	**<0.001**			**−**7.16	3.35	0.03
Spiritual/religious counselor			8.54	0.39			**<**0.0001	7.06	0.35			**<**0.0001
No need	453	0.94			–	–	–			–	–	–
Unmet need	31	0.06			1.48	3.35	0.66			**−**5.03	3.79	0.19
Financial advice			8.53	0.39			**<**0.0001	7.06	0.35			**<**0.0001
No need	407	0.84			–	–	–			–	–	–
Unmet need	77	0.16			0.09	2.29	0.97			**−**3.51	2.58	0.17

*Each model adjusted for age, sex, race/ethnicity, education, marital status, lacking health insurance, cancer type, cancer stage, treatment type, current treatment status, comorbidity, and symptoms.

*^#^Reference group, no need*.

*Significant unmet service needs (*p* < 0.01) are bolded*.

**Table 3:** Multiple linear regression models* examining unmet service needs and SF-12 health-related quality of life summary scores.

Unmet needs^#^	Physical Component Summary							Mental Component Summary				
	*N* = 484	%	*F*	*R*^2^	Beta	SE	*p*	*F*	*R*^2^	Beta	SE	*p*
*Any unmet service need*			8.24	0.38			<0.0001	6.11	0.32			<0.0001
No	312	0.64			–	–	–			–	–	–
Yes	172	0.36			−0.48	0.87	0.58			−**5.79**	**1.05**	<**0.0001**
*Individual unmet service needs*												
Nurse in home			8.23	0.38			<0.0001	4.94	0.27			<0.0001
No need	474	0.98			–	–	–			–	–	–
Unmet need	10	0.02			−1.13	2.72	0.68			−4.09	3.39	0.2284
Support group			8.24	0.38			<0.0001	5.79	0.30			<0.0001
No need	417	0.86			–	–	–			–	–	–
Unmet need	67	0.14			−0.55	1.17	0.64			−**6.76**	**1.43**	<**0.0001**
Mental health professional			8.29	0.39			<0.0001	7.17	0.35			<0.0001
No need	410	0.85			–	–	–			**–**	**–**	**–**
Unmet need	74	0.15			1.21	1.09	0.2642			−**9.65**	**1.28**	<**0.0001**
Physical/occupational therapist			8.57	0.39			<0.0001	5.09	0.28			<0.0001
No need	440	0.91			–	–	–			–	–	–
Unmet need	44	0.09			−**3.76**	**1.40**	**0.007**			−3.99	1.76	0.02
Pain management expert			9.06	0.41			<0.0001	5.04	0.28			<0.0001
No need	442	0.91			**–**	**–**	**–**			**–**	**–**	**–**
Unmet need	42	0.09			−**5.85**	**1.40**	<**0.0001**			−3.46	1.77	0.05
Spiritual/religious counselor			8.49	0.39			<0.0001	5.43	0.29			<0.0001
No need	453	0.94			**–**	**–**	**–**			**–**	**–**	**–**
Unmet need	31	0.06			3.77	1.60	0.02			−**7.30**	**1.98**	<**0.001**
Financial advice			8.28	0.39			<0.0001	4.99	0.27			<0.0001
No need	407	0.84			–	–	–			–	–	–
Unmet need	77	0.16			1.18	1.09	0.2789			−2.22	1.36	0.10

*Each model adjusted for age, sex, race/ethnicity, education, marital status, lacking health insurance, cancer type, cancer stage, treatment type, current treatment status, comorbidity, and symptoms.

*^#^Reference group, no need*.

*Significant unmet service needs (*p* < 0.01) are bolded*.

Table 2 presents PedsQL outcomes for individual service needs. Results for the Total score (overall HRQOL) showed that having a need for social support groups, mental health services, physical/occupational therapy, or pain management services was associated with worse overall HRQOL (all *p*’s < 0.0001). AYA cancer survivors who indicated need of a support group (*p* < 0.0001), mental health professional (*p* < 0.0001), or physical/occupational therapy (*p* < 0.01) reported more fatigue than those who did not report those needs. Worse physical functioning was associated with unmet needs related to participation in a support group (*p* = 0.002) or needs for physical or occupational therapy (*p* = 0.002), or a pain management expert (*p* = 0.001). Lower scores on emotional functioning were associated with needing a support group, a mental health professional, a physical or occupational therapist, or to see a spiritual/religious counselor (all *p*’s < 0.0001). Worse social functioning was associated with needing a support group (*p* = 0.0001), a mental health professional (*p* < 0.0001), a physical or occupational therapist (*p* = 0.002), or pain management services (*p* = 0.001). Finally, unmet needs associated with worse work/school functioning were associated with needing a support group (*p* < 0.0001), mental health services (*p* < 0.0001) or physical/occupational therapy (*p* = 0.009).

Results for SF-12 outcomes and individual service needs (Table 3) indicated that unmet need regarding physical/occupational therapy or pain management services were associated with worse overall physical health on the PCS (*p* < 0.01 and *p* < 0.0001, respectively). On the MCS, our adjusted models indicated that needing a support group, professional mental health services, or needing to see a spiritual/religious counselor, were associated with worse overall mental health (*p*’s < 0.001).

## Discussion

In our study of recently diagnosed AYA cancer survivors, we found that having unmet service needs was strongly associated with lower HRQOL. Furthermore, we identified high levels of unmet service needs in this population, with 35% (*n* = 172) of survivors reporting at least one service need (Keegan et al., 2012). Individual service needs ranged from approximately 15% for financial (*n* = 77) or mental health professional (*n* = 74) services to 2% (*n* = 10) for nursing services in the home. Several specific service needs were associated with HRQOL outcomes and even having one unmet service need was associated with decrements in overall HRQOL, physical, emotional, and social functioning, fatigue, and work/school functioning, as well as overall mental health. Specific needs to see a physical or occupational therapist, a mental health professional, a pain management expert, spiritual/religious counselor, or to participate in a support group were all associated with worse HRQOL domains. Needing mental health services had the strongest associations with worse HRQOL outcomes, while the need for physical or occupational therapy was most consistently associated with poorer functioning across HRQOL domains. Overall, this study extends previous research on unmet service needs (Zebrack, 2008; Keegan et al., 2012; Zebrack et al., 2013) and identifies a critical need to increase services for recent AYA cancer survivors as a means for improving both quality of life and functioning during a highly transitional period of life.

A prominent finding of our study was that having unmet needs for mental health professional services had the strongest associations with outcomes (worse: overall HRQOL, overall mental health, fatigue, emotional functioning, social functioning and work/school functioning), consistent with a recent review (Kahalley et al., 2012). Our finding that 15% of AYA survivors have unmet mental health needs is lower than a study of 215 recently diagnosed AYA cancer patients, where unmet mental health needs ranged from 13.4% in 14- to 19-year-olds to 38.5% of 30- to 39-year-olds (Zebrack et al., 2013), but higher than needs in adult cancer survivors nationally (11.7%) (Hewitt and Rowland, 2002). Individuals indicating a need for support groups reported worse HRQOL on the same domains as those having unmet mental health needs, and also on the physical functioning subscale. However, individuals endorsing needs for mental health and those endorsing support group services differed somewhat, as the correlation between these two service needs was modest (*r* = 0.47; data not shown).Though not surprising that needing mental health services or support groups are associated with worse emotional or social functioning, they are also associated with fatigue and school/work functioning, both of which can have detrimental effects on everyday life activities and adjustment back to normal routines. Studies of pediatric and young adult cancer survivors show that cancer and its treatment have a strong negative impact on work/school (Nagarajan et al., 2003; Parsons et al., 2012); the current study suggests that having better access to mental health support services, or finding ways to increase AYAs’ service utilization, has the potential to partially ameliorate this impact.

While increasing awareness and promoting the use of mental health services to AYA cancer survivors may improve HRQOL, the United States is currently experiencing a severe national shortage of mental health providers, particularly for adolescent psychiatry (Thomas and Holzer, 2006; Masri et al., 2008). Moreover, until recently, mental illness was a criterion that could be used by insurers to deny coverage under the “pre-existing condition” clause (Hyde, 2010). However, under the Affordable Care Act, mental health services will be covered as part of the essential benefits package. As a result, future studies should examine how these policies influence the accessibility and affordability of mental health services for AYA cancer survivors as well as their impact on HRQOL.

Another strong finding in the current paper was that having unmet needs for physical/occupational therapy was associated with poorer outcomes across all HRQOL domains examined except mental health. Though only 9% of the AYA participants in the current study reported unmet physical/occupational therapy service needs, other studies of adults with cancer have reported much a higher prevalence of 30–43% (Thorsen et al., 2011; Holm et al., 2012). Despite a growing appreciation for comprehensive rehabilitation services for cancer survivors (Alfano et al., 2012), physical and occupational therapy utilization rates are likely lower than optimal among AYAs. Young adult survivors of childhood cancers have been found to have low utilization rates of physical therapy and chiropractic use (Montgomery et al., 2011). Future studies should consider the large impact that physical and occupational therapy can have on a variety of health outcomes in AYA, beyond physical functioning.

Our finding that unmet pain management needs were associated with worse overall HRQOL, physical and social functioning are consistent with observations in adult populations. Pain is among the most common cancer symptoms, and a meta-analysis has shown that 33–70% of adult cancer patients report pain symptoms ranging from treatment-related pain to lingering pain in survivorship (Van Den Beuken-Van Everdingen et al., 2007; Valeberg et al., 2008). When pain is unaddressed patients and survivors can experience a negative impact on a range of physical and psychosocial outcomes, such as diminished range of motion and mobility, depressive symptoms, ability to focus, and reduced coping with day to day activities (Institute of Medicine, 2008; Green et al., 2011). While there are estimates that 80–90% of cancer-related pain can be controlled by medication (Stjernsward and Teoh, 1990), barriers limit the ability to identify a need for pain management during cancer treatment or survivorship. Previous research has identified a range of psychosocial barriers that may limit help-seeking related to pain in AYAs such as a fear of addiction to pain medications and concern that social activities may be restricted if pain were reported (Ameringer, 2010). To address some of these concerns, several studies in pediatric and adolescent cancer patients have suggested that complementary and alternative medicine approaches, including acupuncture, meditation, massage, or aromatherapy, may be effective in reducing procedural pain and patient distress (Landier and Tse, 2010). Indeed, Zebrack et al. (2013) reported that 30% of AYAs reported unmet need for complementary and alternative health approaches. However, to fully address pain in the AYA population, an open dialog between providers and patients should be encouraged to identify the most effective approaches to measuring, discussing, and alleviating cancer-related pain.

Our study also showed that unmet needs for religious/spiritual counseling was associated with worse overall mental health and emotional functioning. Prior research suggests that spirituality is associated with better well-being among cancer patients (see Visser et al., 2010 for a review). It is possible that those experiencing the greatest emotional and overall mental health deficits are looking for ways to ameliorate the negative impact of their cancer on their emotional and mental health, with spiritual/religious counseling being one potential avenue. Alternatively, the side effects and logistics regarding cancer treatment may have disrupted usual participation in spiritual or religious services. In either case, there is a need to better provide spiritual counseling to AYA cancer patients who need it.

Results from this study highlight the need to make developmentally appropriate interventions available and accessible to AYAs with cancer. For example, there may be at-risk subgroups by developmental stage related to age at diagnosis, financial independence, employment and insurance status, among other areas. In recent years, AYA-focused clinical programs have developed (Ferrari et al., 2010), and AYA cancer guidelines for clinical practice were released in 2012 from The National Comprehensive Cancer Network (NCCN, 2012) that included a focus on physical and psychosocial issues. Specific recommendations have been made regarding addressing unmet service needs for AYAs with cancer to improve psychosocial outcomes (Zebrack et al., 2009; Kurtz and Abrams, 2010; D’Agostino et al., 2011), and the kinds of psychosocial, work/school, and financial support services that would be appropriate to this age group have been identified (Zebrack and Isaacson, 2012). One important way to promote appropriate supportive care is to ensure that care providers are making necessary referrals (Kahalley et al., 2012) or to specifically train oncology staff to help navigate or provide relevant care (Turner et al., 2009).

The current study’s findings must be considered within the context of its limitations. Although this is the largest population-based study of AYA cancer survivors to date, and the only study to examine associations between support service needs and HRQOL, the design is cross-sectional, relies on self-report of service use, and examines a select set of services. Furthermore, we were not able to examine details about any service programs offered to survivors in the study, and whether unmet services were due to lack of availability, access, or poor communication about available services. Our modest response rate suggests the possibility of a biased sample; however, no major differences were found on demographic and other characteristics between those who did or did not enroll in the study (Harlan et al., 2011). Additionally, we recognize that the age distribution of the current study (15–39 years at diagnosis) is wide and that the cancer types represented are diverse, such that there may be either developmental or disease-specific differences that we were underpowered to explore in detail. We recognize the importance of exploring issues of independence and developmental stages in future research. In particular, age may not be a precise proxy, as there are many independent or mature adults in their 20s, and there are many individuals in their 30s who are living at home or financially supported by parents – which should be independently examined in future research. However, the intention of the AYA HOPE study was to identify broad areas for future research, and the current study helps provide an indication of potential topics for future study. In particular, our study showed important relationships between specific unmet service needs and physical, emotional, and work/school functioning, highlighting mental health needs, physical or occupational therapy needs, and need for better pain management.

In conclusion, the current study suggests that AYAs with cancer have unmet supportive care service needs and that these needs are associated with decrements in HRQOL. In-depth studies are necessary to tease apart issues regarding availability and access to these service needs, and to determine whether they are being offered routinely or there are subsets of individuals not receiving appropriate care. Future research should examine developmentally appropriate, relevant practices for improving access to services that have been shown to adversely impact HRQOL, particularly mental health, physical/occupational therapy, and pain management services.

## Conflict of Interest Statement

The authors declare that the research was conducted in the absence of any commercial or financial relationships that could be construed as a potential conflict of interest.

## References

[B1] Albritton K. Bleyer W. A. (2003). The management of cancer in the older adolescent. Eur. J. Cancer 39, 2584–2599 10.1016/j.ejca.2003.09.013 14642921

[B2] Alfano C. M. Ganz P. A. Rowland J. H. Hahn E. E. (2012). Cancer survivorship and cancer rehabilitation: revitalizing the link. J. Clin. Oncol. 30, 904–906 10.1200/JCO.2011.36.4109 22355063

[B3] Ameringer S. (2010). Barriers to pain management among adolescents with cancer. Pain Manag. Nurs. 11, 224–233 10.1016/j.pmn.2009.05.006 21095597 PMC3011937

[B4] Arora N. K. Hamilton A. S. Potosky A. L. Rowland J. H. Aziz N. M. Bellizzi K. M. (2007). Population-based survivorship research using cancer registries: a study of non-Hodgkin’s lymphoma survivors. J. Cancer Surviv. 1, 49–63 10.1007/s11764-007-0004-3 18648945

[B5] Clinton-McHarg T. Carey M. Sanson-Fisher R. Shakeshaft A. Rainbird K. (2010). Measuring the psychosocial health of adolescent and young adult (AYA) cancer survivors: a critical review. Health Qual. Life Outcomes 8, 25 10.1186/1477-7525-8-25 20205922 PMC2850329

[B6] D’Agostino N. M. Penney A. Zebrack B. (2011). Providing developmentally appropriate psychosocial care to adolescent and young adult cancer survivors. Cancer 117, 2329–2334 10.1002/cncr.26043 21523754

[B7] Dyson G. J. Thompson K. Palmer S. Thomas D. M. Schofield P. (2012). The relationship between unmet needs and distress amongst young people with cancer. Support. Care Cancer 20, 75–85 10.1007/s00520-010-1059-7 21311915

[B8] Ewing J. E. King M. T. Smith N. F. (2009). Validation of modified forms of the PedsQL generic core scales and cancer module scales for adolescents and young adults (AYA) with cancer or a blood disorder. Qual. Life Res. 18, 231–244 10.1007/s11136-008-9424-4 19165624

[B9] Fernandez C. V. Barr R. D. (2006). Adolescents and young adults with cancer: an orphaned population. Paediatr. Child Health 11, 103–106 19030262 10.1093/pch/11.2.103 PMC2435335

[B10] Ferrari A. Thomas D. Franklin A. R. Hayes-Lattin B. M. Mascarin M. Van Der Graaf W. (2010). Starting an adolescent and young adult program: some success stories and some obstacles to overcome. J. Clin. Oncol. 28, 4850–4857 10.1200/JCO.2009.25.0803 20479411

[B11] Girgis A. Boyes A. Sanson-Fisher R. W. Burrows S. (2000). Perceived needs of women diagnosed with breast cancer: rural versus urban location. Aust. N. Z. J. Public Health 24, 166–173 10.1111/j.1467-842X.2000.tb00137.x 10790936

[B12] Green C. R. Hart-Johnson T. Loeffler D. R. (2011). Cancer-related chronic pain: examining quality of life in diverse cancer survivors. Cancer 117, 1994–2003 10.1002/cncr.25761 21509777

[B13] Hall A. E. Boyes A. W. Bowman J. Walsh R. A. James E. L. Girgis A. (2012). Young adult cancer survivors’ psychosocial well-being: a cross-sectional study assessing quality of life, unmet needs, and health behaviors. Support. Care Cancer 20, 1333–1341 10.1007/s00520-012-1434-7 21720746

[B14] Harlan L. C. Lynch C. F. Keegan T. H. Hamilton A. S. Wu X. C. Kato I. (2011). Recruitment and follow-up of adolescent and young adult cancer survivors: the AYA HOPE Study. J. Cancer Surviv. 5, 305–314 10.1007/s11764-011-0173-y 21274648 PMC3159756

[B15] Hewitt M. Rowland J. H. (2002). Mental health service use among adult cancer survivors: analyses of the National Health Interview Survey. J. Clin. Oncol. 20, 4581–4590 10.1200/JCO.2002.03.077 12454116

[B16] Holm L. V. Hansen D. G. Johansen C. Vedsted P. Larsen P. V. Kragstrup J. (2012). Participation in cancer rehabilitation and unmet needs: a population-based cohort study. Support. Care Cancer 20, 2913–2924 10.1007/s00520-012-1420-0 22415608 PMC3461205

[B17] Hyde P. (2010). The Affordable Care Act & Mental Health: An Update. Available at: http://www.healthcare.gov/blog/2010/08/mentalhealthupdate.html [accessed December 5, 2012].

[B18] Institute of Medicine. (2008). Cancer Care for the Whole Patient: Meeting Psychosocial Health Needs. Washington, DC: The National Academies Press 20669419

[B19] Kahalley L. S. Wilson S. J. Tyc V. L. Conklin H. M. Hudson M. M. Wu S. (2012). Are the psychological needs of adolescent survivors of pediatric cancer adequately identified and treated? Psychooncology. 22, 447–458 22278930 10.1002/pon.3021 PMC3360126

[B20] Keegan T. H. Lichtensztajn D. Y. Kato I. Kent E. E. Wu X. C. West M. M. (2012). Unmet adolescent and young adult cancer survivors information and service needs: a population-based cancer registry study. J. Cancer Surviv. 6, 239–250 10.1007/s11764-012-0219-9 22457219 PMC3433596

[B21] Kent E. E. Smith A. W. Keegan T. H. M. Lynch C. F. Wu X. C. Hamilton A. S. (in press). Talking about cancer and meeting peer survivors: social information needs of adolescents and young adults diagnosed with cancer. J. Adolesc. Young Adult Oncol. 10.1089/jayao.2012.0029 PMC3684139 23781400

[B22] Kurtz B. P. Abrams A. N. (2010). Psychiatric aspects of pediatric cancer. Child Adolesc. Psychiatr. Clin. N. Am. 19, 401–421 10.1016/j.chc.2010.01.009 20478507

[B23] Landier W. Tse A. M. (2010). Use of complementary and alternative medical interventions for the management of procedure-related pain, anxiety, and distress in pediatric oncology: an integrative review. J. Pediatr. Nurs. 25, 566–579 10.1016/j.pedn.2010.01.009 21035021 PMC4944826

[B24] Masri G. Cuffe S. Edwards L. (2008). Mental health issues in adolescents and young adults. Northeast Fla. Med. 59, 24–25

[B25] Montgomery M. Huang S. Cox C. L. Leisenring W. M. Oeffinger K. C. Hudson M. M. (2011). Physical therapy and chiropractic use among childhood cancer survivors with chronic disease: impact on health-related quality of life. J. Cancer Surviv. 5, 73–81 10.1007/s11764-010-0151-9 20922492 PMC3062253

[B26] Nagarajan R. Neglia J. P. Clohisy D. R. Yasui Y. Greenberg M. Hudson M. (2003). Education, employment, insurance, and marital status among 694 survivors of pediatric lower extremity bone tumors: a report from the childhood cancer survivor study. Cancer 97, 2554–2564 10.1002/cncr.11363 12733155

[B27] National Cancer Institute. (2011). *A Snapshot of Adolescent and Young Adult Cancers* Available at: http://www.cancer.gov/PublishedContent/Files/aboutnci/servingpeople/snapshots/2011_AYA_snapshot.508.pdf [accessed December 17, 2012].

[B28] Parsons H. M. Harlan L. C. Lynch C. F. Hamilton A. S. Wu X. C. Kato I. (2012). Impact of cancer on work and education among adolescent and young adult cancer survivors. J. Clin. Oncol. 30, 2393–2400 10.1200/JCO.2011.39.6333 22614977 PMC3675694

[B29] Sanson-Fisher R. Girgis A. Boyes A. Bonevski B. Burton L. Cook P. (2000). The unmet supportive care needs of patients with cancer. Supportive Care Review Group. Cancer 88, 226–237 10.1002/(SICI)1097-0142(20000101)88:1<226::AID-CNCR30>3.0.CO;2-P 10618627

[B30] Smith A. W. Bellizzi K. M. Keegan T. H. M. Zebrack B. Chen V. W. Neale A. V. (in press). Health-related quality of life of adolescent and young adult cancer patients in the United States: the AYA HOPE study. 10.1200/JCO.2012.47.3173 PMC3731979 23650427

[B31] Stava C. J. Lopez A. Vassilopoulou-Sellin R. (2006). Health profiles of younger and older breast cancer survivors. Cancer 107, 1752–1759 10.1002/cncr.22200 16967441

[B32] Stjernsward J. Teoh N. (1990). “The scope of the cancer pain problem,” in Advances in Pain Research and Therapy (Proceedings of the Second International Congress on Cancer Pain), eds Foley K. Bonica J. Ventafridda V. (New York: Raven Press), 7–12

[B33] Thomas C. R. Holzer C. E. III (2006). The continuing shortage of child and adolescent psychiatrists. J. Am. Acad. Child Adolesc. Psychiatry 45, 1023–1031 10.1097/01.chi.0000184928.33799.60 16840879

[B34] Thorsen L. Gjerset G. M. Loge J. H. Kiserud C. E. Skovlund E. Flotten T. (2011). Cancer patients’ needs for rehabilitation services. Acta Oncol. 50, 212–222 10.3109/0284186X.2010.531050 21231783

[B35] Turner J. Clavarino A. Butow P. Yates P. Hargraves M. Connors V. (2009). Enhancing the capacity of oncology nurses to provide supportive care for parents with advanced cancer: evaluation of an educational intervention. Eur. J. Cancer 45, 1798–1806 10.1016/j.ejca.2009.02.023 19329294

[B36] Valeberg B. T. Rustoen T. Bjordal K. Hanestad B. R. Paul S. Miaskowski C. (2008). Self-reported prevalence, etiology, and characteristics of pain in oncology outpatients. Eur. J. Pain 12, 582–590 10.1016/j.ejpain.2007.09.004 18023377

[B37] Van Den Beuken-Van Everdingen M. H. De Rijke J. M. Kessels A. G. Schouten H. C. Van Kleef M. Patijn J. (2007). Prevalence of pain in patients with cancer: a systematic review of the past 40 years. Ann. Oncol. 18, 1437–1449 10.1093/annonc/mdm056 17355955

[B38] Varni J. W. Limbers C. A. (2008). The PedsQL Multidimensional Fatigue Scale in young adults: feasibility, reliability and validity in a University student population. Qual. Life Res. 17, 105–114 10.1007/s11136-008-9389-3 18027106

[B39] Varni J. W. Limbers C. A. (2009). The PedsQL 4.0 Generic Core Scales Young Adult Version: feasibility, reliability and validity in a university student population. J. Health Psychol. 14, 611–622 10.1177/1359105309103580 19383661

[B40] Visser A. Garssen B. Vingerhoets A. (2010). Spirituality and well-being in cancer patients: a review. Psychooncology 19, 565–572 19916163 10.1002/pon.1626

[B41] Ware J. Kosinski M. Turner-Bowker D. Gandek B. (2002). How to Score Version 2 of the SF-12 Health Survey (With a Supplement Documenting Version 1). Lincoln, RI: QualityMetric Incorporated

[B42] Ware J. E. Jr. Kosinski M. Turner-Bowker D. M. Sundaram M. Gandek B. Maruish M. E. (2009). User’s Manual for the SF12v2 Health Survey. Lincoln, RI: QualityMetric

[B43] Zebrack B. (2008). Information and service needs for young adult cancer patients. Support. Care Cancer 16, 1353–1360 10.1007/s00520-008-0435-z 18386075

[B44] Zebrack B. Hamilton R. Smith A. W. (2009). Psychosocial outcomes and service use among young adults with cancer. Semin. Oncol. 36, 468–477 10.1053/j.seminoncol.2009.07.003 19835742

[B45] Zebrack B. Isaacson S. (2012). Psychosocial care of adolescent and young adult patients with cancer and survivors. J. Clin. Oncol. 30, 1221–1226 10.1200/JCO.2011.39.5467 22412147

[B46] Zebrack B. J. Block R. Hayes-Lattin B. Embry L. Aguilar C. Meeske K. A. (2013). Psychosocial service use and unmet need among recently diagnosed adolescent and young adult cancer patients. Cancer 119, 201–214 10.1002/cncr.27713 22744865

